# Complete remission after pembrolizumab monotherapy in a non-small cell lung cancer patient with PD-L1 negative, high tumor mutational burden, and positive tumor-infiltrating lymphocytes: A case report

**DOI:** 10.1097/MD.0000000000040369

**Published:** 2024-12-06

**Authors:** Suoni Li, Jiequn Ma, Jie Bai, Zheng Zhao

**Affiliations:** a Department of Oncology, Shaanxi Provincial Tumor Hospital, Xi’an, Shaanxi, China.

**Keywords:** immune checkpoint inhibitor, non-small cell lung cancer, pembrolizumab, programmed cell death ligand-1, tumor mutation burden, tumor-infiltrating lymphocytes

## Abstract

**Rationale:**

Immune checkpoint inhibitors have been used to treat cancer patients. Non-small cell lung cancer (NSCLC) patients with a high expression level of programmed cell death ligand-1 (PD-L1) could benefit from immune checkpoint inhibitor monotherapy. However, treating NSCLC patients with PD-L1 negative is still a clinical challenge. The utilization of new-type tumor markers as predictive indicators of therapeutic efficacy, with the aim of guiding clinical medication strategies, has emerged as a paramount focus of clinical investigation and interest.

**Patient concerns and diagnoses:**

We reported a 72-year-old male with cough diagnosed as poorly differentiated metastatic lung adenocarcinoma (cT3N2M1, stage IV). He tested negative for driver gene mutations, and PD-L1 negative (＜1%), but a high tumor mutational burden (30.9 and 39.1 mutations/Mb in the lung tissue and blood, respectively), and positive tumor-infiltrating lymphocytes.

**Interventions:**

The patient received pembrolizumab monotherapy.

**Outcomes:**

After 8 treatment cycles over 5 months, repeat examinations showed significantly reduced lung mass and circulating tumor DNA abundance. The patient reached clinical complete remission and had long-term survival with no significant adverse events.

**Lessons:**

A comprehensive evaluation of multiple tumor biomarkers should be considered in NSCLC patients. Pembrolizumab monotherapy could benefit NSCLC patients with negative driver genes, PD-L1 negative, a high tumor mutational burden, and positive tumor-infiltrating lymphocytes.

## 1. Introduction

Lung cancer ranks 2nd in incidence and 1st in mortality among all tumors in the world.^[1]^ Non-small cell lung cancer (NSCLC) accounts for 85% of all types of lung cancer.^[2]^ In recent years, immune checkpoint inhibitors (ICIs) have been recognized as a breakthrough in cancer therapy. However, ICIs showed the most benefits in NSCLC patients with a high expression level of the programmed cell death ligand-1 (PD-L1).^[3]^ Therefore, pembrolizumab, one of the ICIs inhibiting PD-L1, was recommended in NSCLC patients with a PD-L1 expression >50%.^[4]^ Whether patients with a low PD-L1 expression level or PD-L1 negative could benefit from pembrolizumab monotherapy is still in question, and whose treatment is a clinical challenge. Here, we describe an elderly patient with metastatic NSCLC with negative driver genes and PD-L1 negative, but a high tumor mutational burden (TMB) and positive tumor-infiltrating lymphocytes (TILs). After comprehensively considering multiple biomarkers, we applied pembrolizumab monotherapy, achieving a satisfactory long-term outcome in this patient. We report and discuss our experience here.

## 2. Case presentation

**Figure 1.**
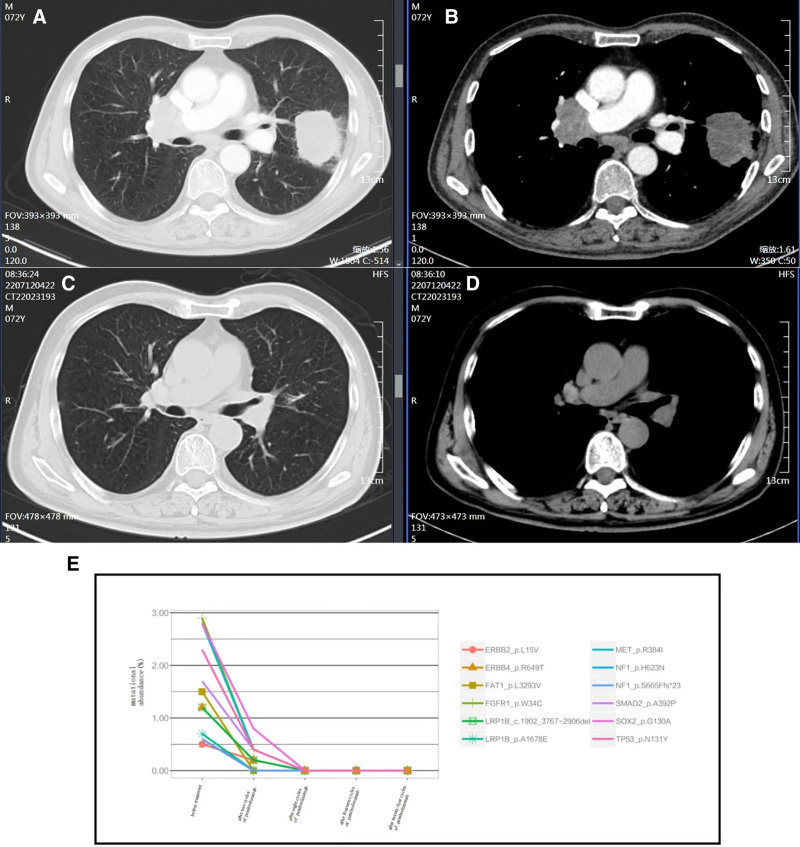
Computed tomography images before and after the pembrolizumab monotherapy. Before the treatment, there were bilateral multiple pulmonary nodules with a mass in the left upper lobe (arrow) (A) and enlarged lymph nodes in the mediastinum and right hilar area (arrows) (B). After the treatment for 8 cycles, the left lung mass disappeared (C), and the lymph nodes in the mediastinum and right hilar area had significantly reduced sizes (D). Circulating tumor DNA abundance in the blood samples decreased significantly after the 2 cycles of the pembrolizumab monotherapy (E).

**Figure 2.**
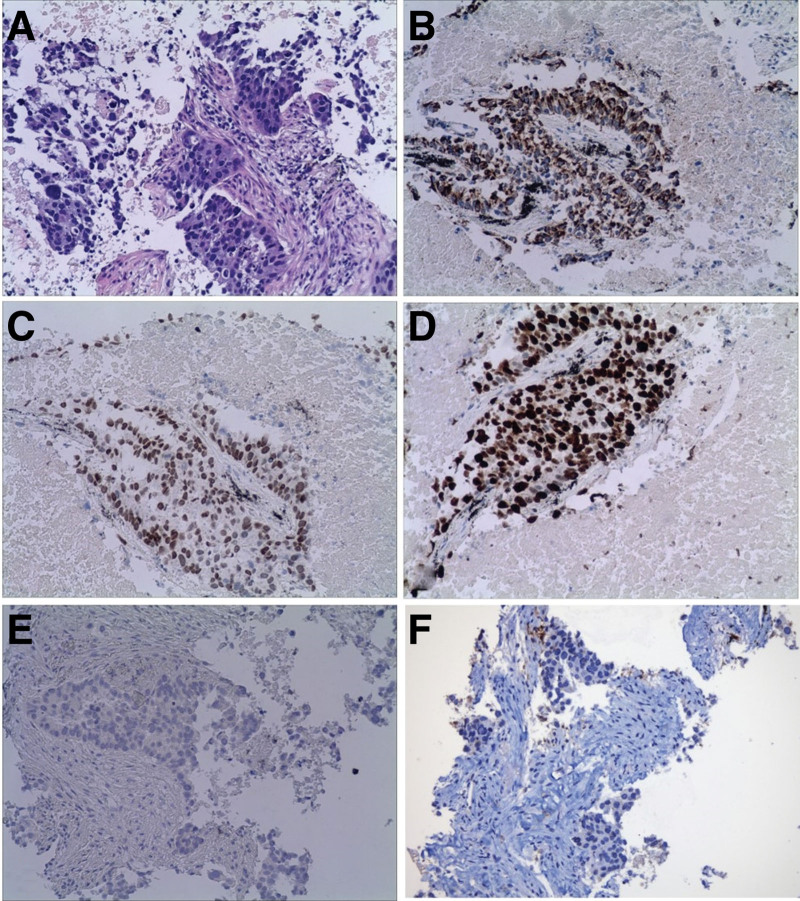
Pathological reports. (A) Hematoxylin and eosin staining show poorly differentiated adenocarcinoma with cells arranged in solid nest-like or small adenoids, atypical nuclei, and necrosis. (B) Positive CK7 cytoplasm. (C) Positive TTF-1 nuclei. (D) Ki-67 differentiation index 90%. (E) Negative VENTANA ALK (D5F3). (F) PD-L1 (Daco 22C3) < 1% expression.

**Figure 3.**
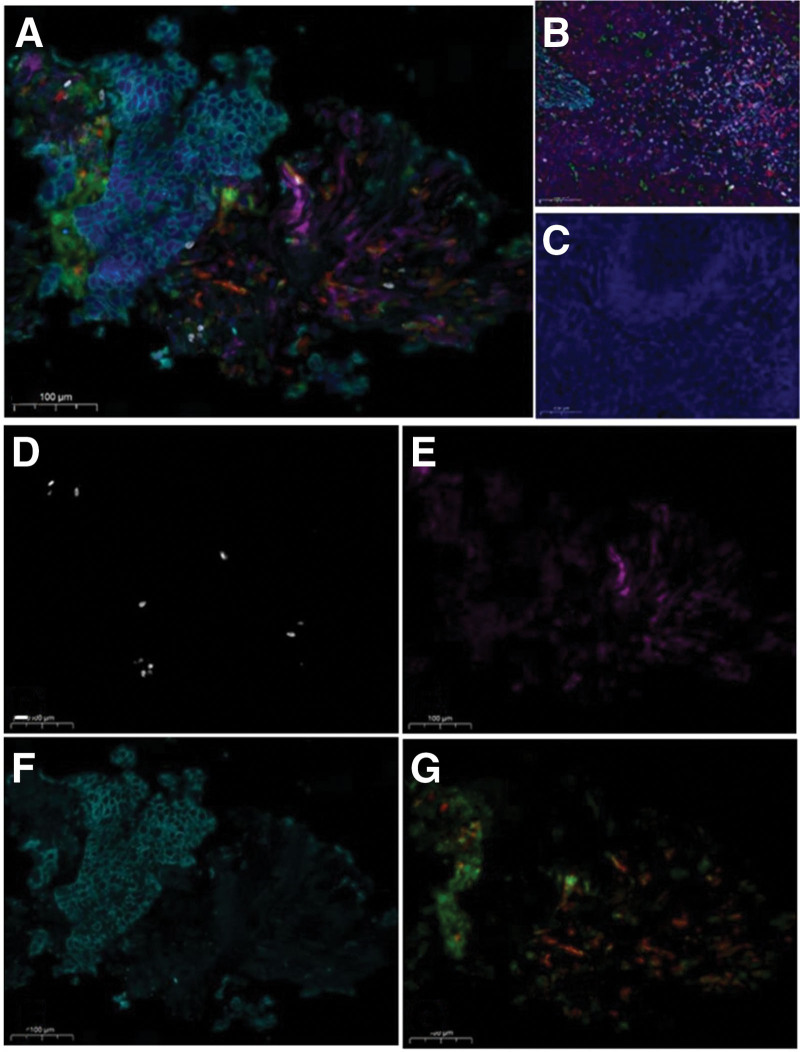
Multiplex fluorescence immunohistochemistry study. (A) Panorama (300X). (B) Positive control (300X). (C) Negative control (300X). (D) CD8 (white). (E) CD56 (purple). (F) PanCK (ching). (G) HLA-DR (red), CD68 (green).

On September 14, 2021, a 72-year-old male presented to our hospital with a progressively worsening cough for 1 month. His mother died of glioma, but he denied any past medical history. His vital signs included a temperature of 36.8 °C, a pulse of 88 beats/min, a respiration rate of 20 breaths/min, and a blood pressure of 125/78 mm Hg. The physical examinations were unremarkable. The laboratory tests showed normal white blood cell count and hemoglobin level. The platelet count was significantly elevated (502 × 10^9^/L). The liver function, kidney function, electrolytes, coagulation panel, and myocardial enzyme were within normal limits. However, tumor marker tests reported high alpha-fetoprotein levels (15.3 ng/mL, normal range 0–5.5 ng/mL) and cytokeratin (CF-211, 15.9 ng/mL, normal range 0–7 ng/mL). The echocardiography showed an ejection fraction of 60%. The electrocardiogram reported sinus rhythm with a complete right bundle branch block. A chest computed tomography (CT) scan showed bilaterally multiple pulmonary nodules with a mass in the left upper lobe (Fig. 1A). There were also enlarged lymph nodes in the mediastinum and right hilar area (Fig. 1B). After obtaining consent from the patient, a CT-guided puncture biopsy of the left lung mass was performed. The pathological study reported poorly differentiated adenocarcinoma, with immunohistochemistry showing CK5/6 (-), P40 (few +), EGFR (sp111) (1+), EGFR (sp125) (+), CK7 (+), TTF-1 (+), Ki-67 (60%+), VENTANA ALK (D5F3) (-), BRAF (-), and PD-L1 (Daco 22C3) < 1% (Fig. 2). Next generation sequencings suggested negative driver genes. The TMB in lung tissue sample and blood sample was 30.9 and 39.1 mutations/Mb, respectively. Multiplex fluorescence immunohistochemistry was used to detect cytotoxic T cells, tumor-associated macrophages, and natural killer (NK) cells. The results showed sparse CD8+ T cells, abundant M2 type tumor promoting tumor-associated macrophages and extensive infiltration of NK cells in the tumor parenchyma and interstitial area (Fig. 3 and Table 1). Further position emission tomography scan showed no lesions outside the thoracic area. The patient was diagnosed with left lung poorly differentiated adenocarcinoma, T3N2M1, stage IV with bilateral intrapulmonary and mediastinal and right hilar lymph node metastases, negative driver gene, PD-L1-/TILs+, and high TMB.

**Table 1:** Results of cell compositions of tumor immune microenvironment by multiplex fluorescence immunohistochemistry study.

Cell types	Markers	Tumor parenchyma		Tumor stroma	
		Numbers (mm^2^)	Positive rate (%)	Numbers (mm^2^)	Positive rate (%)
Cytotoxic T cells	CD8+	6.2	0.2	24.2	1.8
Tumor-associated macrophages	CD68+HLA−DR+	31.2	0.7	119.8	8.9
	CD68+HLA−DR−	74.9	1.8	192.6	14.3
Natural killer cells	CD56bright	21.8	0.5	88.9	6.6
	CD56dim	96.7	2.3	216.8	16.1

The patient’s PD-L1 expression was negative. According to the guidelines,^[5]^ his treatment regimen should be immunotherapy combined with platinum-containing chemotherapy. Despite negative PD-L1 expression, few CD8+ T cells, and a high number of M2 type tumor promoting tumor-associated macrophages, this patient still had a high TMB. Additionally, there was significant infiltration of NK cells in the tumor tissue and surrounding area. This suggested that the patient could be an excellent candidate for immunotherapy. After comprehensive evaluations of his PD-L1, TILs, and TMB status, we decided to initiate the pembrolizumab monotherapy (200 mg once every 3 weeks) for him. The patient received 2 cycles of treatment. The repeat chest computed tomography (CT) scan showed that the mass in the left lung was slightly smaller, but other lung nodules were slightly larger, without significant changes in the lymph nodes in the mediastinum. However, the TMB and circulating tumor DNA (ctDNA) abundance in the blood samples decreased significantly after the treatment (Fig. 1E), suggesting the effectiveness of the pembrolizumab monotherapy. Therefore, pembrolizumab was administrated for additional 2 cycles. A repeat chest CT scan reported significantly reduced sizes of the left lung mass and bilateral lung nodules. The patient was continued on the pembrolizumab monotherapy and was followed up in the clinic. Five months after initiating the pembrolizumab treatment, a repeat chest CT scan showed that the left lung mass and bilateral lung nodules had also significantly reduced and nearly disappeared (Fig. 1C and D). The blood TMB could not be detected, and ctDNA was rapidly cleared (Fig. 1E). This patient is still under pembrolizumab monotherapy and followed up in the clinic. He did not report any adverse events during the pembrolizumab monotherapy. His NSCLC was considered a clinical complete remission according to the RECIST criteria.^[6]^

## 3. Discussion

This case report describes an NSCLC patient with negative driver genes and PD-L1 negative, but a high TMB and TILs+. He was treated with pembrolizumab monotherapy, leading to clinical complete remission and long-term survival with no significant adverse events. In advanced NSCLC patients with the negative driver gene, single or combined ICIs treatment has become the preferred regimen recommended by many guidelines. In clinical practice, the challenge is finding the appropriate candidate for ICI treatment. There were many studies on the biomarkers, such as PD-L1, TMB, and TILs, as well as tumor immune microenvironment classification and tumor immune dysfunction and rejection (TIDE) scoring system, to predict the treatment response to ICIs.

The programmed cell death receptor-1 (PD-1)/PD-L1 is an important pathway that mediates tumor cell immune escape and promotes tumor cell proliferation.^[7]^ The ICIs could block the PD-1/PD-L1 pathway to suppress tumor growth. The KEYNOTE-024 study showed the efficacy of pembrolizumab monotherapy in advanced NSCLC patients with PD-L1 expression ≥50%. However, the KEYNOTE-407 research showed that patients with PD-L1 expression <1% could still benefit from the ICIs treatment. The patient we reported was PD-L1 negative and responded well to the pembrolizumab monotherapy. Therefore, we considered that PD-L1 as a biomarker has a certain value in predicting the immunotherapeutic efficacy in NSCLC patients. However, its expression level was not always correlated with clinical efficacy. The underlying reason might be related to tumor heterogeneity, instability of PD-L1 expression, and other detection and scoring methods. Recently, deglycosylated PD-L1 was applied in PD-L1 detection, which improved the detection probability of PD-L1,^[8]^ and might become a new indicator for predicting the ICI treatment efficacy. In addition, a retrospective study using ICIs to treat NSCLC patients showed that the increased PD-L1 level in macrophages was associated with a better survival chance, suggesting that the PD-L1 level in the macrophages might be used to predict the ICI treatment response.^[9]^ The study result should be confirmed.

TMB can be tested both in tissue samples (tissue TMB) and blood samples (blood TMB). Previous studies have reported controversial results on the relationship between the TMB value and the efficiency of ICI treatment response.^[10–12]^ Checkmate227 study found that compared with TMB-L (TMB＜10 muts/Mb) patients, TMB-H (TMB ≥ 10 muts/Mb) patients had better PFS and OS benefits when using immunotherapy. Checkmate026 study based on PD-L1 stratification did not find the difference between PFS and OS between the 2 groups. But based on TMB stratification, it was found that TMB-H patients had higher PFS and ORR, but there was no difference in OS. However, KEYNOTE201 study did not find the correlation between the expression of TMB and the efficacy of ICIs. So, the predictive value of TMB for immunotherapy is still being explored. In this case the patient had a high TMB value and response well to the ICI treatment. The various responses of the tumor with high TMB value to the ICI treatments might be due to the inconsistent detection methods, tumor heterogeneity, and different thresholds for defining high and low levels of TMB.

Tumor microenvironment refers to the living environment of tumors, which mainly consists of tumor cells, surrounding blood vessels, extracellular matrix, immune cells, tumor related fibroblasts, various cytokines, chemokines, etc. It is a key factor affecting tumor growth and metastasis. The tumor immune microenvironment classification is based on the tumor PD-L1 expression status and the presence of TILs.^[13]^ At present, some classification and scoring systems have been proposed to predict the response to ICI treatment. NSCLC patients with the PD-L1+/TILs+ type might be more likely to benefit from ICI therapy. Our patient could be classified as the PD-L1−/TILs+ type, but still achieved a satisfactory response to the ICI monotherapy. The TIDE scoring system integrated the results of 33,197 cancer samples from 189 studies. It developed a computational framework based on the characteristic genes of T cell dysfunction and T cell rejection analysis. The final results showed that the TIDE score was better than other biomarkers, including TMB, PD-L1, and IFN γ, in predicting outcomes of the ICI treatment.^[14]^ The authors suggested that the TIDE scoring system could be used to predict the ICI treatment prognosis. More studies are required on this classification and scoring systems.

## 4. Conclusion

Immunotherapy with ICIs has significantly improved our treatment success in patients with NSCLC. The treatment challenge is to find the appropriate candidates for ICI therapy. A combination of multiple biomarkers may be used to better predict treatment responses. Here, we show that an NSCLS patient with negative driver genes, negative PD-L1 expression, a high tumor mutational burden, and positive TILs could still show satisfactory long-term responses to the pembrolizumab monotherapy. The treatment responses should be closely monitored, for example, by the ctDNA abundance and repeat image evaluations. However, this article is a case report, and further clinical studies with larger sample sizes are needed to verify the conclusions drawn from this case report.

## Author contributions

**Data curation:** Jie Bai.

**Project administration:** Zheng Zhao.

**Writing – original draft:** Suoni Li.

**Writing – review & editing:** Jiequn Ma.
